# Correction: Women’s experiences of and satisfaction with childbirth: Development and validation of a measurement scale for low- and middle-income countries

**DOI:** 10.1371/journal.pone.0331720

**Published:** 2025-09-04

**Authors:** The PLOS One Staff

In the Study instrument subsection of the Materials and methods, the Appendix 1 hyperlink in the third sentence is not working. The correct third sentence is: The instrument was developed in English, then translated to French, Spanish, Thai, and Vietnamese to pilot and for data collection (translations available in Appendix 1).

In the Results section, the hyperlink in the second sentence of the second paragraph is not working. The correct second sentence is: Appendix 2 shows item scores (mean, standard deviation, median, and correlation coefficient) for the QD-BES items and dimensions.

In the Results section, the hyperlink in the fifth sentence of the third paragraph is not working. The correct fifth sentence is: All items demonstrated heterogeneous floor and ceiling effects across all women (Appendix 2).

In the Validity subsection of the Results, the hyperlink in the second sentence of the first paragraph is not working. The correct second sentence is: The results showed good model fit, with a Comparative Fit Index of 0.92 to 0.96 across countries (good quality model), Incremental Fit Index of 0.92 to 0.97 across countries (adequate model fit), Root Mean Square Error of Approximation of 0.07 to 0.24 across countries (acceptable model fit), and Standardized Root Mean Square Residual of 0.07 across countries (acceptable model fit) (Appendix 3).

The publisher apologizes for the errors.
