# Supplementary material for: Women’s experiences of and satisfaction with childbirth: Development and validation of a measurement scale for low- and middle-income countries
Source: PLoS One. 2025 May 27;20(5):e0322132. doi: 10.1371/journal.pone.0322132 (PMC12112374; doi:10.1371/journal.pone.0322132)
Supplement: Attachment — Submitted filename: Response to reviewers_02-28-2025mb.docx [file pone.0322132.s003.docx]

Dear Editors,

Thank you for the editorial and peer review feedback, which we believe has strengthened our paper. Please see the point-by-point response to peer review and editorial feedback below.

Best wishes,

Meghan Bohren, on behalf of the authors

| Reviewer/editor comments | Author response |
| --- | --- |
| *Reviewer #1* |  |
| This is a rigorous manuscript and makes a significant contribution to measuring person-centred outcomes in maternity care globally.  My main suggestion is around framing potential use of the tool. Given the broad nature of item questions, the authors could highlighting the importance of balance with qualitative feedback mechanisms within services, to understand the nuances of women’s and providers' experiences and what is required to improve services. | Thank you for the positive feedback on our paper.  We have added to the discussion section (page 21) about the importance of qualitative feedback:  *“Quantitative evaluations of experiences of and satisfaction with maternity care should also be balanced with qualitative assessment, via in-depth interviews and/or focus group discussions with women and health workers. These qualitative approaches can help provide more nuanced understanding of women’s perspectives, and may help elucidate how and why both positive and negative experiences happen.”* |
| In the Abstract, it would seem important to mention the tool was developed in the context of improving decision-making for caesarean section and measuring program outcomes. | We have revised the abstract to include a brief overview of the QUALI-DEC study. |
| *Reviewer #2* |  |
| Your effort in writing the article is clear and distinctive, and here are some points that need to be developed to make the research better, especially in the field of measurement and questionnaires and their validity. There is a clear deficiency in the statistical tests used, and they are not sufficient to judge the validity of the scale alone. There are several statistical procedures that must be performed, including:   1. Cronbach's alpha 2. Guttman coefficient 3. Half-split coefficient 4. Principal Component Analysis 5. Exploratory and confirmatory factor analysis   Use the AMOS program to study these tests because it is the best in that  And it provides you with the charts that illustrate the necessary components of the scales you are developing  You should strongly review the steps for measuring validity used in this article as a comprehensive example It is recommended to follow the same steps so that your work is at the highest scientific level of accuracy and comprehensiveness: <https://link.springer.com/article/10.1186/s12909-024-05731-5> | We thank the reviewer for their comment. We used SPSS instead of AMOS software, and are confident that it would lead to comparable results. The comparability of SPSS and AMOS is illustrated in the following paper: <https://pubs.sciepub.com/amp/5/3/2/>.  Concerning the statistical procedures suggested by the reviewer, we note that we implemented 4 out of 5 mentioned in the reviewer’s comment. As you can see in our methods and results sections:   - Cronbach alphas are mentioned in the methods section and reported in Table 2 - We implemented exploratory factor analysis using principal component analysis with a promax rotation as mentioned in the data methods-analysis section. The results are available in Figure 3 and Appendix 2. - A confirmatory factor analysis was also carried out and allowed us to report IFI, CFI, RMSEA and SRMR as can be seen in the validity section   A priori, we decided not to compute Guttman coefficient, because its use relies on hypotheses that are not consistent with our analysis approach. Indeed, the Guttman coefficient is especially relevant for monotonic scales, based on item response theory. We agree that in our case, the use of Guttman coefficient would have been relevant to assess each item’s contribution to the global scale. However, our analyses already assessed each item’s contribution by computing Cronbach’s alpha, and by checking the inter-item correlation matrix.  We also agree that the use of the half-split coefficient is a relevant approach to test for internal consistency. However, we used Cronbach’s alpha to test for internal consistency, because the result would be similar to that from the use of half split coefficient (e.g., as can be seen in the following paper <https://onlinelibrary.wiley.com/doi/full/10.1155/2014/742863>). In addition, we computed intraclass correlation coefficients to ensure the reliability and the stability of the scale across groups.  In order to ensure the robustness of our analytic approach for validity testing, we have followed the steps described in the following article <https://www.frontiersin.org/journals/public-health/articles/10.3389/fpubh.2018.00149/full> |
| Finally, the method of calculating the sample size should also be explained in more detail. | We have included a detailed description of the sample size method:  *“Sample size estimation was based on the expected difference in satisfaction scores between the period before and after the intervention. The required number of women needed for a “before-after” cross-section design was a total of 470 women per country (60 women per hospital) to ensure 90% statistical power to detect an effect size of 0.3 standard deviations or greater in satisfaction scores. The sample size calculation would ensure sufficient power for country-specific comparisons. The calculation accounted for the clustered nature of the data by hospitals with a design effect of 2. Assuming a 10% non-response rate and 10% of ineligible women, we aimed to approach 2,256 women total: 564 women per country, 71 women per hospital.”*  No revision made. |
| *Editorial feedback* |  |
| 1.  Please ensure that your manuscript meets PLOS ONE's style requirements, including those for file naming. The PLOS ONE style templates can be found at  [https://journals.plos.org/plosone/s/file?id=wjVg/PLOSOne_formatting_sample_main_body.pdf](https://track.editorialmanager.com/CL0/https:%2F%2Fjournals.plos.org%2Fplosone%2Fs%2Ffile%3Fid=wjVg%2FPLOSOne_formatting_sample_main_body.pdf/1/010f0193f5ab2a37-4a2a637b-a2ef-452b-a57a-73e4733cbe32-000000/RPOQbtOSh2hoeCfxM_mpDRwKiUqD8M3tLePGGZlmr0Y=189)  and  [https://journals.plos.org/plosone/s/file?id=ba62/PLOSOne_formatting_sample_title_authors_affiliations.pdf](https://track.editorialmanager.com/CL0/https:%2F%2Fjournals.plos.org%2Fplosone%2Fs%2Ffile%3Fid=ba62%2FPLOSOne_formatting_sample_title_authors_affiliations.pdf/1/010f0193f5ab2a37-4a2a637b-a2ef-452b-a57a-73e4733cbe32-000000/A5UAB7kB3tRFQZvDLcVZOjQhUfz1LJGBFbwnsn5YIXQ=189) | We confirm our manuscript meets PLOS One style requirements. |
| 2. Please include a complete copy of PLOS’ questionnaire on inclusivity in global research in your revised manuscript. Our policy for research in this area aims to improve transparency in the reporting of research performed outside of researchers’ own country or community. The policy applies to researchers who have travelled to a different country to conduct research, research with Indigenous populations or their lands, and research on cultural artefacts. The questionnaire can also be requested at the journal’s discretion for any other submissions, even if these conditions are not met. Please find more information on the policy and a link to download a blank copy of the questionnaire here: <https://journals.plos.org/plosone/s/best-practices-in-research-reporting>. Please upload a completed version of your questionnaire as Supporting Information when you resubmit your manuscript. | The ’inclusivity in global research’ form is completed and uploaded. |
| 3. Thank you for stating the following in your Competing Interests section: “None declared.” Please complete your Competing Interests on the online submission form to state any Competing Interests. If you have no competing interests, please state "The authors have declared that no competing interests exist.", as detailed online in our guide for authors at <http://journals.plos.org/plosone/s/submit-now>   This information should be included in your cover letter; we will change the online submission form on your behalf. | We confirm that the authors have declared that no competing interests exist. We have updated this in the cover letter online. |
| 4. In this instance it seems there may be acceptable restrictions in place that prevent the public sharing of your minimal data. However, in line with our goal of ensuring long-term data availability to all interested researchers, PLOS’ Data Policy states that authors cannot be the sole named individuals responsible for ensuring data access (<http://journals.plos.org/plosone/s/data-availability#loc-acceptable-data-sharing-methods>).  Data requests to a non-author institutional point of contact, such as a data access or ethics committee, helps guarantee long term stability and availability of data. Providing interested researchers with a durable point of contact ensures data will be accessible even if an author changes email addresses, institutions, or becomes unavailable to answer requests.  Before we proceed with your manuscript, please also provide non-author contact information (phone/email/hyperlink) for a data access committee, ethics committee, or other institutional body to which data requests may be sent. If no institutional body is available to respond to requests for your minimal data, please consider if there any institutional representatives who did not collaborate in the study, and are not listed as authors on the manuscript, who would be able to hold the data and respond to external requests for data access? If so, please provide their contact information (i.e., email address). Please also provide details on how you will ensure persistent or long-term data storage and availability. | The data are currently stored on a secure, password-protected server (REDCap), accessible only to the QUALI-DEC team research team.  We note that this paper presents an interim analysis that is part of a larger QUALI-DEC project – an implementation trial. At the end of the QUALI-DEC trial (July 2025), the anonymized dataset will be freely accessible on the QUALI-DEC Zenodo platform:  [https://zenodo.org/communities/qualidec/records?q=&l=list&p=1&s=10&sort=newest](https://url.au.m.mimecastprotect.com/s/FTsoC1WZKqh6jrOJNuLfyTVU55B?domain=zenodo.org)  This will include the survey data presented in this analysis. Could you please advise if this is sufficient to respond to the data access and accessibility concern raised? |
| 5. When completing the data availability statement of the submission form, you indicated that you will make your data available on acceptance. We strongly recommend all authors decide on a data sharing plan before acceptance, as the process can be lengthy and hold up publication timelines. Please note that, though access restrictions are acceptable now, your entire data will need to be made freely accessible if your manuscript is accepted for publication. This policy applies to all data except where public deposition would breach compliance with the protocol approved by your research ethics board. If you are unable to adhere to our open data policy, please kindly revise your statement to explain your reasoning and we will seek the editor's input on an exemption. Please be assured that, once you have provided your new statement, the assessment of your exemption will not hold up the peer review process. | Please see response in #4 above. |
| 6. Your ethics statement should only appear in the Methods section of your manuscript. If your ethics statement is written in any section besides the Methods, please move it to the Methods section and delete it from any other section. Please ensure that your ethics statement is included in your manuscript, as the ethics statement entered into the online submission form will not be published alongside your manuscript. | We have moved the ethics section to the Methods section of the manuscript and deleted from the other section. |

Additional editorial feedback 26 February 2025

| Editorial comment | Author response |
| --- | --- |
| 1. In the online submission form you indicate that your data is not available for proprietary reasons and have provided a contact point for accessing this data. Please note that your current contact point is a co-author on this manuscript. According to our Data Policy, the contact point must not be an author on the manuscript and must be an institutional contact, ideally not an individual. Please revise your data statement to a non-author institutional point of contact, such as a data access or ethics committee, and send this to us via return email. Please also include contact information for the third party organization, and please include the full citation of where the data can be found. | We have revised the ‘Availability of data and materials’ section of the paper to state below text, and responded via email to this request.  The datasets used and/or analyzed during the current study will be available from the completion of the QUALI-DEC trial in July 2025 via the QUALI-DEC study Zenodo platform: https://zenodo.org/communities/qualidec/records?q=&l=list&p=1&s=10&sort=newest. Requests regarding data use should be directed to: qualidecproject@gmail.com |
| 2. Please upload a Response to Reviewers letter which should include a point by point response to each of the points made by the Editor and / or Reviewers. (This should be uploaded as a 'Response to Reviewers' file type.) Please follow this link for more information: <http://blogs.PLOS.org/everyone/2011/05/10/how-to-submit-your-revised-manuscript/> | A response to reviewers letter has been uploaded to Editorial manager |
| 3. Can you please upload an additional copy of your revised manuscript that does not contain any tracked changes or highlighting as your main article file. This will be used in the production process if your manuscript is accepted. Please amend the file type for the file showing your changes to Revised Manuscript w/tracked changes. Please follow this link for more information: <http://blogs.PLOS.org/everyone/2011/05/10/how-to-submit-your-revised-manuscript/> | A clean version of the manuscript has been uploaded to Editorial manager |
